# Feasibility of Introducing a Prehabilitation Program into the Care of Gynecological Oncology Patients—A Single Institution Experience

**DOI:** 10.3390/cancers16051013

**Published:** 2024-02-29

**Authors:** Joëlle Dhanis, Dieuwke Strijker, Luuk D. Drager, Maaike van Ham, Cornelis J. H. M. van Laarhoven, Johanna M. A. Pijnenborg, Anke Smits, Baukje van den Heuvel

**Affiliations:** 1 Department of Obstetrics and Gynecology, Radboud University Medical Center, Geert Grooteplein Zuid 10, 6525 GA Nijmegen, The Netherlands; maaike.vanham@radboudumc.nl (M.v.H.); anke.smits@radboudumc.nl (A.S.); 2 Department of Operating Rooms, Radboud University Medical Center, Geert Grooteplein Zuid 10, 6525 GA Nijmegen, The Netherlands; 3 Department of Surgery, Radboud University Medical Center, Geert Grooteplein Zuid 10, 6525 GA Nijmegen, The Netherlandskees.vanlaarhoven@radboudumc.nl (C.J.H.M.v.L.)

**Keywords:** multimodal prehabilitation, gynecological cancer, endometrial cancer, ovarian cancer, vulvar cancer, implementation, adherence, recruitment, safety

## Abstract

**Simple Summary:**

Surgery is the cornerstone in the treatment of most gynecological cancers and is associated with significant risks. Gynecological cancer patients are generally elderly, overweight, and have a sedentary lifestyle, which are all factors associated with surgical complications. Prehabilitation entails the optimizing of a patient’s condition prior to surgery, and has been introduced with the goal of improving postoperative outcomes and recovery. A prehabilitation program has been implemented at an academic hospital in The Netherlands and comprises a physical exercise intervention, nutritional intervention, psychological intervention, and an intoxication cessation program. In this study, we assessed the feasibility of this program in terms of recruitment, adherence, and safety of implementation into the standard care of gynecological oncology patients. The principal finding was that the implementation of a prehabilitation program for gynecological oncology patients is feasible, with high recruitment and adherence rates. In addition, we did not encounter any major adverse events, suggesting its safety.

**Abstract:**

Prehabilitation is an upcoming strategy to optimize patient’s functional capacity, nutritional status, and psychosocial well-being in order to reduce surgical complications and enhance recovery. This study aims to assess the feasibility of implementing a multimodal prehabilitation program into the standard care of gynecological oncology patients at an academic hospital in terms of recruitment, adherence, and safety, which were assessed by the number of patients eligible, recruitment rate, participation rate, and adherence to individual modalities. Data were derived from the F4S PREHAB trial, a single-center stepped-wedge trial implementing a multimodal prehabilitation program among various surgical specialties. All patients undergoing elective surgery as part of treatment for ovarian, uterine, and vulvar cancer at the Radboudumc, an academic hospital in The Netherlands, between May 2022 and September 2023 were considered eligible for the F4S PREHAB trial and, consequently, were included in this cohort study. The multimodal prehabilitation program comprised a physical exercise intervention, nutritional intervention, psychological intervention, and an intoxication cessation program. A total of 152 patients were eligible and approached for participation of which 111 consented to participate, resulting in a recruitment rate of 73%. Participants attended an average of six exercise sessions and adhered to 85% of possible training sessions. Respectively, 93% and 98% of participants adhered to the prescribed daily protein and vitamin suppletion. Ten participants were referred to a psychologist and completed consultations. Out of nine active smokers, two managed to quit smoking. A total of 59% adhered to alcohol cessation advice. No adverse events were reported. This study demonstrates that introducing a multimodal prehabilitation program into the standard care of gynecological oncology patients is feasible in terms of recruitment and adherence, with no serious adverse events.

## 1. Introduction

It is well known that many gynecological cancer patients struggle with an unhealthy lifestyle, which predisposes them to both disease development and increased risk of treatment-related morbidity [1,2,3]. Endometrial and vulvar cancer patients are typically characterized by advancing age, obesity, and a sedentary lifestyle, with endometrial cancer showing the strongest association with body mass index (BMI) of all cancers [4,5,6,7,8,9]. Up to 81% of all endometrial cancer patients are obese (BMI > 30 kg/m^2^), and the majority do not meet exercise recommendations [10,11,12]. Ovarian cancer patients often present with malnourishment due to tumor growth, which leads to protein depletion, and only 21% of patients adhere to exercise guidelines [1,13,14].

Surgery is the cornerstone in the treatment of most gynecological cancers. Despite variation in surgical treatment across different tumor sites, surgery is associated with significant risks. Postoperative complications have been reported in a significant number of patients with gynecological cancers, which can be partly attributed to patient characteristics including advanced age, obesity, and a sedentary lifestyle [15,16,17]. In addition, these patients are at risk of a worse prognosis and poorer quality of life if they are unable to achieve healthier lifestyle behaviors [1,18,19,20,21,22]. Prehabilitation programs have been suggested to improve both surgical outcomes and quality of life through optimization of individual, modifiable risk factors prior to surgery [23]. Recently, multimodal prehabilitation programs for colorectal and other abdominal cancer surgery yielded favorable outcomes by significantly reducing surgical complications and the length of hospital stays [24,25,26,27,28,29]. Based on similarities in risk factors addressed by multimodal prehabilitation among various cancer sites [30,31], prehabilitation is expected to potentially improve postoperative outcomes for patients with gynecological malignancy. However, within gynecological cancer surgery, prehabilitation remains in its infancy; only a few studies have been performed and these are hampered by poor methodological quality, small study population sizes, and heterogeneity of interventions. The current evidence is insufficient to fully support the feasibility of prehabilitation for this population, and no definitive conclusions have been drawn regarding adherence and postoperative outcomes [32].

A multimodal prehabilitation program has been introduced as an integrative part of the surgical pathway as part of a stepped-wedge trial (F4S PREHAB trial) at the Radboudumc, an academic hospital in The Netherlands, including the Gynecological Oncology Department. Following the current lack of evidence, we report our institutional experience with the feasibility of implementing this prehabilitation program in terms of recruitment, adherence, and safety in the standard care of gynecological cancer patients.

## 2. Materials and Methods

### 2.1. Study Design

This was a prospective cohort study of a subgroup of the F4S PREHAB trial—a single-center stepped-wedge trial implementing a multimodal prehabilitation program into the care of patients in various surgical pathways at the Radboudumc, The Netherlands. The protocol has been approved by the Medical Research Ethics Committee (MREC) Oost-Nederland (NL73777.091.20). The Gynecological Oncology Department was included in the F4S PREHAB trial and is a tertiary gynecological oncology center in The Netherlands. Patients in the intervention group participated in the multimodal prehabilitation program alongside standard preoperative care consisting of general recommendations according to national guidelines [33].

### 2.2. Study Population and Recruitment

All patients undergoing elective surgery for (suspected) ovarian, uterine, or vulvar cancer at the Radboudumc between May 2022 and September 2023, and who were considered for the F4S PREHAB trial, were included in this cohort study. Inclusion and exclusion criteria for the F4S PREHAB trial have been previously defined [34]. Patients presenting at the outpatient gynecologic oncology clinic were screened for eligibility. When eligible, an appointment was made with the prehabilitation team. Patients were given information prior to the appointment, where informed consent was obtained.

### 2.3. Intervention

The intervention consisted of a multimodal prehabilitation program including an exercise, nutritional, and psychological component, and intoxication cessation, which participants received in addition to standard perioperative care. The program has been previously described [34]. The length of the program was determined by the time between diagnosis and surgery and was aimed to encompass a minimum of three weeks.

The exercise component consisted of three-weekly individual supervised sessions with a physiotherapist in the community. The sessions included both endurance training and resistance training [35]. To identify risks of adverse cardiovascular events during exercise, the program was preceded by a screening of participants using the Exercise Preparticipation Health Screening Questionnaire for Exercise Professionals by the American College of Sports Medicine [36]. Patients who were identified to be at risk were referred to a cardiologist or pulmonologist to undergo additional tests to assess whether undergoing high-intensity training as part of prehabilitation was deemed safe. Workload was based on the results of the individual physical fitness assessment, which included the Steep Ramp test and indirect 1-Repetition Maximum (1-RM) test (based on leg press). On days without supervised training, participants were instructed to perform low-intensity aerobic exercise (for example walking, cycling, or swimming) for 60 min a day.

For the nutritional component, participants were screened using the Patient-Generated Subjective Global Assessment (PG-SGA) [37]. Dietary advice was given, aiming for optimal energy and a daily protein intake of 1.5 g/kg body weight based on BMI and three-day food diaries. If BMI was lower than 20 kg/m^2^ or higher than 30 kg/m^2^, body weight was corrected to 20 kg/m^2^ and 27.5 kg/m^2^, respectively [38]. Participants received high-quality whey protein shakes (Nutri Whey™ Isolate, Friesland Campina, Wageningen, The Netherlands) containing 30 grams of whey protein and 20 micrograms of vitamin D on a daily basis, with an additional shake following supervised exercise. Furthermore, participants were instructed to take multivitamin supplementation (fifty percent of the daily recommendation) to improve possible vitamin deficiencies.

The Hospital Anxiety and Depression Scale (HADS) was used to identify psychological factors that may adversely affect postoperative outcomes among participants [39]. Participants at risk (score ≥ 15) were referred to a psychologist if they were interested in receiving counseling to improve coping strategies and anxiety regarding their diagnosis and surgery.

Participants who were actively smoking were offered a referral to an external smoking cessation program (SineFuma, Breda, The Netherlands), which included intensive counseling and nicotine replacement therapy. In addition, all patients were advised to stop alcohol consumption.

### 2.4. Data Collection

Data were extracted from the F4S PREHAB trial and electronic patient files. Baseline characteristics collected were age at diagnosis, previous medical history, Charlson Comorbidity Index (CCI) [40], Eastern Cooperative Oncology Group (ECOG) performance status, BMI, smoking status, and alcohol consumption. Clinical characteristics included gynecological cancer type, ASA score, and type of surgery. In addition, daily protein intake, estimated by a dietician based on daily intake reports, and weekly exercise were collected at baseline and post-intervention. Data regarding adherence were collected from the physiotherapist and self-reported questionnaires. Serious and adverse events related to the intervention were also collected from patient files, reported by the physiotherapist, nutritionist, or patient.

### 2.5. Study Outcome

The primary aim of this study was to assess the feasibility of implementing a multimodal prehabilitation program into the standard care of gynecological oncology patients in terms of 1. recruitment, 2. adherence, and 3. safety. This was subsequently assessed by 1. the number of patients eligible, recruitment rate (percentage of eligible patients included), and participation rate (percentage of eligible patients actually participating in the study); 2. adherence to the program’s individual components; and 3. the occurrence of serious and adverse events related to the intervention.

Adherence rates were calculated according to the following descriptions:

(1) Exercise intervention
  - Supervised program: The percentage of completed training sessions out of possible training sessions in the optimal training period (the time between the first training session and surgery). The interval between patient inclusion and the first training session is excluded due to the logistical factor of referral to the physiotherapist and not patient adherence. The number of possible training sessions in the optimal training period entails three sessions per seven days.
  - Low-intensity exercise advice: The percentage of completed low-intensity exercise sessions out of the number of instructed low-intensity exercise sessions per week, with the maximum being four, as participants were instructed to execute this on days without supervised training.

(2) Nutritional intervention
  - Protein supplementation: The percentage of days on which protein supplementation was completed out of the number of days that protein supplementation was prescribed. The maximum number is fixed at seven as participants were instructed to take daily supplementation.
  - Vitamin supplementation: The percentage of completed doses of vitamin supplements out of prescribed doses (multivitamin supplementation was prescribed daily).

(3) Psychological intervention: The psychological intervention comprised sessions with a medical psychologist to receive counseling to improve coping strategies and anxiety regarding their diagnosis and surgery. This component was designed as a consulting and supporting intervention.
(4) Intoxication cessation
  - Smoking: The number of participants successfully quitting smoking out of all active smokers.
  - Alcohol: The number of participants successfully quitting alcohol consumption out of all participants consuming alcohol.

The following adherence rates were calculated based on patient-reported adherence: low-intensity exercise advice, protein and vitamin supplementation, and smoking and alcohol cessation.

Secondary outcomes included factors associated with non-participation or non-adherence (defined as discontinuing the program), changes in daily protein intake, and adherence to the National Exercise Guidelines [33]. Factors analyzed for association included age, weight, BMI, histology, stage, ECOG performance status, comorbidities, smoking, daily protein intake, and adherence to national exercise guidelines.

### 2.6. Statistical Analysis

All data were analyzed using IBM SPSS Statistics Version 25 [41]. Continuous outcomes are presented as the mean and standard deviation and the median and interquartile range (IQR)/range and were compared using the Mann–Whitney U test. Categorical data are presented as frequencies and proportions and were compared using Chi-square and Fisher’s exact tests, where appropriate. Differences in continuous outcomes before and after prehabilitation were compared using the Wilcoxon Signed Rank test.

## 3. Results

### 3.1. Recruitment

**Figure 1:**
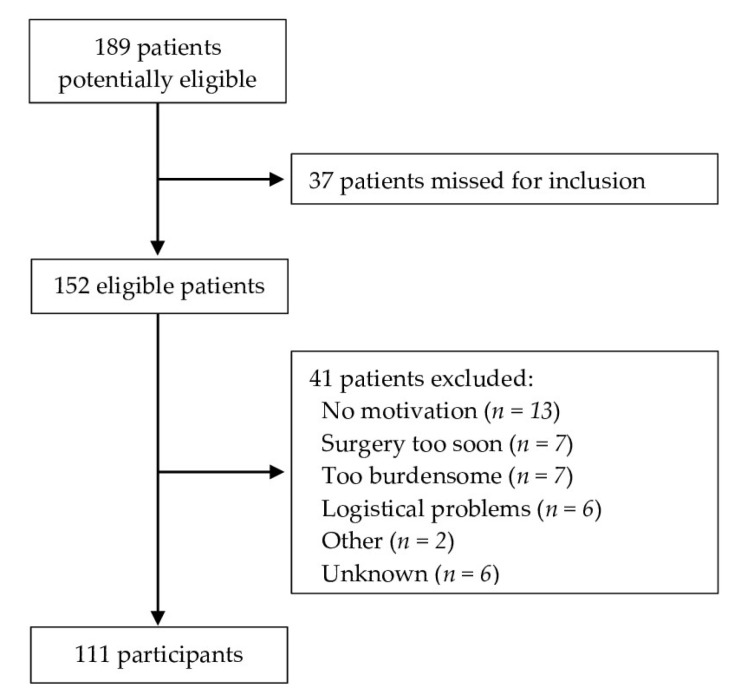
Flow chart for inclusion and exclusion.

A total of 189 patients were planned for elective surgery for (suspected) ovarian, uterine, and vulvar cancer between May 2022 and September 2023 and were eligible for participation in the F4S PREHAB trial. A total of 37 eligible patients were not approached for inclusion in the first months of implementing the F4S PREHAB trial due to triaging errors and internal referrals, leaving a total of 152 patients who were approached. A total of 111 patients (73%) consented to participate in the study (recruitment rate) (Figure 1). Reasons for not participating included the following: lack of motivation (*n* = 13), the interval between inclusion and surgery was expected to be too short due to rescheduling of the surgery and patient’s perspectives (*n* = 7), the program being perceived as too burdensome by the patient (*n* = 7), and logistical problems in terms of planning (*n* = 6).

### 3.2. Patient Demographics and Clinical Characteristics

Baseline characteristics are shown in Table 1. The median age was 66 years (range 24–87 years). The median BMI was 27.2 kg/m^2^ (range 20–48 kg/m^2^). Seventy-two percent of participants had an ECOG performance status of 0. Most participants had a CCI ≤ 1 (77%). The most prevalent comorbidities were cardiovascular comorbidities (44%). Nine participants were active smokers (8%).

Uterine cancer (43%) was the most prevalent cancer diagnosis followed by ovarian (36%) and vulvar cancer (20%). Up to 45% of ovarian cancer participants received neoadjuvant chemotherapy prior to surgery. One participant with endometrial cancer received neoadjuvant chemotherapy prior to surgery because of pulmonary metastasis. Surgical procedures are specified in Table 1. The majority of ovarian cancer patients had debulking surgery (interval: 46%, *n* = 16; primary: 11%, *n* = 4). Almost all patients with uterine cancer received a hysterectomy with bilateral salpingo-oophorectomy (98%, *n* = 39). All but one vulvar cancer patient underwent a vulvectomy. There were no significant differences in the baseline regarding age, BMI, CCI, histology, and smoking between patients participating and those who declined to participate.

### 3.3. Adherence

**Figure 2:**
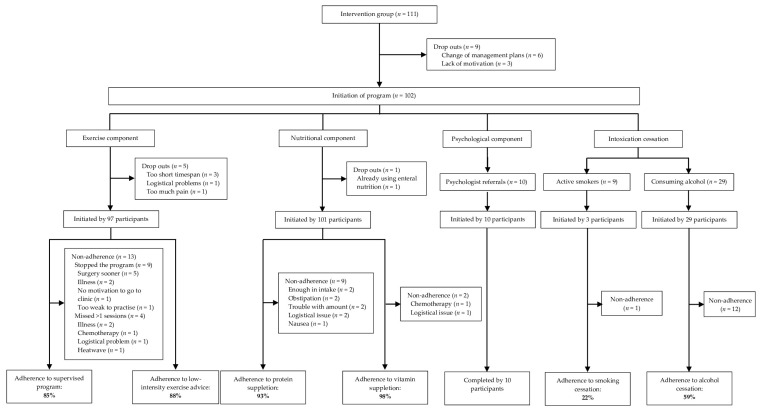
Flow chart for participation and adherence.

After the first consultation, a total of nine participants did not start any of the components due to changes in surgical management (*n* = 6) or a loss of motivation for the program (*n* = 3), resulting in a participation rate of 67% (102/152; see Figure 2). The exercise component was initiated by 97 participants (95%) while the nutritional component was initiated by 101 participants (99%). The median duration of the program (patient inclusion until preoperative assessment) was twenty days (IQR 15–26; range 7–62). Participation and adherence rates are visualized in Figure 2.

#### 3.3.1. Exercise Component

The average number of exercise sessions was six (IQR 4–8; range 0–10) and the average duration of the optimal training period was fourteen days (IQR 8–19; range 1–44), matching the intended three sessions a week. Participants adhered to 85% of possible training sessions (Table 2). Nine participants discontinued the exercise program because surgery was rescheduled to an earlier date (*n* = 5), illness (*n* = 2), lack of motivation to go to the physiotherapy clinic (*n* = 1), or feeling too weak to practice (*n* = 1). The majority of the remaining 37 participants missed a single session out of all possible sessions in the optimal training period with the main reasons being logistical problems and feeling unwell, although reasons were infrequently documented. For eleven participants, the intensity of training was adjusted, by lowering the intensity for ten participants and increasing it for one.

Regarding the low-intensity exercise advice, the median number of sessions per week was four (IQR 3–4; range 0–4) and participants adhered to 88% of all possible sessions. Data regarding low-intensity exercise were missing for 15 participants (Table 2).

Prior to the intervention, 73% of all participants adhered to the national exercise guidelines (> 150 min a week of mild aerobic exercise). After the intervention, there was a significant improvement in reported exercise (*p* = 0.013), with 88% of participants adhering to the national guidelines. Data regarding daily exercise were missing for 29 participants.

#### 3.3.2. Nutritional Component

The nutritional intervention comprised two components—vitamin supplementation and protein supplementation. The median number of servings per week was 7 (IQR 7–7; range 0–7) for both protein supplementation and vitamin supplementation, resulting in an adherence rate of 93% and 96%, respectively (Table 3). Reasons for not completing daily protein supplementation included nausea (*n* = 2) and obstipation (*n* = 2). Further reasons are specified in Figure 2.

The average number of dietician consultations was two (IQR 2–2; range 1–6) per participant. The estimated daily crude protein intake increased significantly from 70 grams per day at baseline to 105 grams per day after prehabilitation (*p* < 0.001). Estimated daily protein intake relative to kilograms of body weight significantly increased by 0.45 grams per kilogram (*p* < 0.001). At the start of the intervention, 11% of participants (*n* = 10) had a daily protein intake corrected for body weight of ≥1.5 g/kg. After the intervention, 59% of the participants reported a daily protein intake of ≥1.5 g/kg (*p* < 0.001).

#### 3.3.3. Psychological Component

Following the assessment using the HADS, ten participants (9%) were referred to a psychologist. Each participant received a single session with the psychologist. No further consultations, therapy, or medication were needed.

#### 3.3.4. Intoxication Cessation

Nine participants (8%) were active smokers, of which five agreed to be referred to the smoking cessation program. Three participants opted to participate in the smoking cessation program, of which two managed to successfully quit smoking and one lessened the number of cigarettes smoked per day, resulting in an adherence rate of 22%. In addition, a total of 29 participants consumed alcohol preoperatively, of which seventeen managed to stop drinking, resulting in an adherence rate of 59%.

### 3.4. Factors Associated with Participation and Adherence

Baseline characteristics including age, weight, BMI, histology, stage, ECOG performance status, comorbidities, smoking, daily protein intake, and adherence to exercise guidelines were assessed for associations with participation in the program and adherence (defined as continuing the program until surgery) to the program’s individual exercise and nutrition components. No factors were significantly associated with participation in the program compared to non-participation, and no factors were significantly associated with adherence to both the exercise and nutritional components nor with adherence to solely the exercise or the nutritional component.

### 3.5. Adverse Events

There were no reports of any serious or adverse events during the program.

## 4. Discussion

This study shows that the implementation of multimodal prehabilitation into the standard care for gynecological oncology patients is feasible in terms of recruitment, adherence, and safety. The recruitment and participation rates were 73% and 67%, respectively. Adherence rates varied per component and ranged from 22% and 59% for smoking and alcohol cessation, respectively, up to 85–88% for the exercise component and 93–98% for the nutritional component. This multimodal prehabilitation program, as part of the F4S PREHAB trial, is the first program that has been introduced as standard care for gynecological oncology patients in The Netherlands.

Within gynecological oncology, few studies assessing adherence to (multimodal) prehabilitation interventions have been performed. Diaz-Feijoo et al. reported equally high rates of participation and adherence to the exercise component of 75% and 87%, respectively; reasons for not participating mainly included a lack of motivation and insufficient time prior to surgery, suggesting that through extensive counseling and possibly adjusted scheduling of the surgery in relation to the prehabilitation program, participation can be optimized [42]. In our population, we observed a relatively high adherence to national exercise guidelines (73%), as previously reported rates vary between 21% and 48% for ovarian and endometrial cancer, respectively [10,11,12,14]. This may have impacted the participation and adherence to the program as participants might be more motivated to participate due to their pre-existing fitness levels. Yet, as adherence was based on ‘self-reporting’ by patients, this might be overestimated. For the nutritional component, comparable adherence rates were found in the current literature, suggesting that a protein-rich diet including supplementation is easily implemented [42,43]. Adherence to the smoking cessation program was low but in accordance with the literature [44]. In addition, the recent literature reports adherence rates to smoking cessation before a gynecological surgery to be 15–30% [45]. Very few studies have been performed to evaluate preoperative alcohol cessation. One trial assessing a comprehensive intervention program showed a quitting rate of 90% [46]. As the adherence to the advice to stop alcohol consumption was 59% in our study, better adherence could be gained via improved counseling that emphasizes its importance. Overall, our reported participation and adherence rates suggest that gynecological oncology patients are motivated for prehabilitation.

In surgical oncology, prehabilitation programs have been assessed more extensively. Particularly in colorectal cancer, prehabilitation programs have shown promising results, with significant reductions in the length of hospital stays and up to a 50% reduction in operative complications [26,29,47,48,49]. The majority of these programs encompass multimodal interventions, with exercise and nutrition being important mainstays of the intervention. For gynecological oncology, the benefits of prehabilitation programs have not yet been established. Our study group recently performed a systematic review supporting the feasibility and safety of a prehabilitation program in gynecological oncology care [32]; seven studies were included, and considerable heterogeneity of interventions and outcome measures was observed. Significant improvements in hospital stay and time to adjuvant treatment without impacting surgical complications were reported [32,42]. In two more recent studies, a significant improvement in nutritional parameters, lower intra-operative transfusion rates, and a shortened time to normal diet were also observed [50,51]. Even though data on the contributions of the individual components are lacking, multimodal prehabilitation shows significant results that are not seen through unimodal prehabilitation. This would suggest that the synergistic effect of multimodal prehabilitation is most beneficial to improve perioperative outcomes.

As we have demonstrated the feasibility of prehabilitation in an academic setting in terms of recruitment, adherence, and safety, future research should focus on assessing the effect on perioperative outcomes in this population and within the different gynecological cancer types. In order to be able to define cut-off values for adherence and determine the specific effect of each component, it is of importance to address adherence to all individual modalities in further research. The assessed participation rate of 67% and the varying adherence rates in our study suggest room for improvement in recruitment and adherence to multimodal prehabilitation programs. Therefore, patient’s perspectives on multimodal prehabilitation need to be further explored using semi-structured interviews or focus groups to gain insight into the barriers and facilitators regarding participation and adherence.

This study is a subgroup analysis of the F4S PREHAB trial, which is one of the first studies implementing a multimodal prehabilitation program into the routine care of gynecological oncology patients. In addition, vulvar cancer patients were also included, which has not been described in other studies. A further strength is that the exercise component consisted of individualized, one-to-one supervised sessions with a local physiotherapist in the community, which are known factors to improve engagement and overcome barriers to participation [52,53].

However, there are some limitations to this study. As this was a single-institution experience of a tertiary gynecological oncology center, applicability to other institutions may be limited. In addition, the feasibility aspects of the prehabilitation program were not primary nor secondary outcomes of the F4S PREHAB trial. However, following the paucity of data for gynecological cancer patients specifically, we do believe in the importance of reporting our institutional experience. Unfortunately, there is no standardized way of calculating adherence to a multimodal program in the literature, and this is particularly challenging for multimodal programs. We defined adherence as the percentage of completed sessions/servings out of all possible sessions/servings. Other studies have defined adherence as successfully fulfilling 80% or more of the possible sessions/servings of one particular modality, disregarding the possible synergistic effect of the different modalities [42]. Therefore, standard regulation of defining adherence to a multimodal prehabilitation program still needs to be determined. Secondly, some patients were missed for inclusion, which may have skewed our results. This was the result of logistical issues accompanying the implementation of a trial and, therefore, was not due to deliberate patient selection. In addition, we observed no adverse events based on reports from physiotherapists, nutritionists, and patients. Although we believe this demonstrates safety in terms of serious adverse events and injuries, we cannot conclude a complete absence of adverse events or other side effects as this was also not an outcome of the F4S PREHAB trial. Lastly, adherence to several of the components was based on patient-reported outcomes, which led to some missing data regarding the fulfillment of weekly unsupervised exercise and alcohol usage.

## 5. Conclusions

This study demonstrates that the implementation of a multimodal prehabilitation program into the standard care for ovarian, endometrial, and vulvar cancer patients is feasible in terms of recruitment and adherence, with high recruitment and participation rates of 73% and 67%, respectively. These rates support patient’s motivations to participate in and complete a prehabilitation program. There was significant variation in the adherence rates per component, ranging from 22% up to 98%, with the main reasons for non-adherence being a change in management plans, feeling unwell, and no motivation. It is of utmost importance to report adherence on every individual modality to be able to determine the influence of each component on the synergistic effects of multimodal prehabilitation. Further research is needed to gain insight into defining and improving adherence and to determine the effectiveness and specific benefits of prehabilitation programs in improving surgical and long-term outcomes for this specific population.

## Figures and Tables

**Table 1:** Baseline characteristics.

Characteristics	Participants *n* = 111 (%)
Age [years], median (IQR; range)	66 (59–75; 24–87)
Weight [kg], median (IQR; range)	76.6 (64.1–87.8; 55–120.8)
BMI [kg/m^2^], *n* (%)	
18.5–25	34 (30.6)
25–30	37 (33.3)
>30	40 (36.0)
Comorbidities, *n* (%)	
None	30 (27.0)
1	35 (31.5)
2	18 (16.2)
>2	28 (25.2)
Charlson Comorbidity Index, *n* (%)	
0–1	85 (76.6)
>1	26 (23.4)
ECOG, *n* (%)	
0	80 (72.1)
1	23 (20.7)
2	7 (6.3)
3	1 (0.9)
Smoking, *n* (%)	
Yes	9 (8.1)
No	102 (91.7)
Alcohol consumption, *n* (%)	
Yes	29 (26.1)
No	52 (46.8)
Unknown	30 (27.1)
ASA score, *n* (%)	
1	5 (4.5)
2	76 (67.5)
3	30 (27.0)
Cancer diagnosis, *n* (%)	
Uterine	48 (43.2)
FIGO I–II	30 (62.6)
FIGO III–IV	14 (29.2)
Benign	4 (8.3)
Ovarian	40 (36.0)
FIGO I–II	14 (35.0)
FIGO III–IV	23 (57.5)
Borderline/teratoma	3 (7.5)
Vulvar	22 (19.8)
FIGO I–II	14 (63.63)
FIGO III–IV	6 (27.2)
DVIN/melanoma	2 (9.1)
Uterine and ovarian	1 (0.9)
Surgical procedure, *n* (%)	
Ovarian cancer	
Debulking	23 (57.5)
Laparoscopic staging	5 (12.5)
Exploratory laparotomy	10 (25.0)
Other	2 (5.0)
Uterine cancer	
Uterus + Adnexa	17 (35.4)
Uterus + Adnexa + SN + LND	30 (62.5)
Other	1 (2.1)
Vulvar cancer	
Vulvectomy	4 (18.2)
Vulvectomy + lymph node surgery	17 (77.3)
Exenterative surgery	1 (4.5)

BMI: Body Mass Index; ECOG: Eastern Cooperative Oncology Group; ASA: American Society of Anesthesiologists; SN: Sentinel node procedure; LND: lymph node dissection.

**Table 2:** Adherence to and completion of the exercise program.

	**Participants (*n* = 97)**
**Supervised training program**	
Possible training sessions, median (IQR; range) ^a^	6 (3–8; 2–19)
Completed training sessions, median (IQR; range) ^b^	6 (3–7; 0–10)
Adherence rate, % ^c^	85%
**Unsupervised training program**	
Possible training sessions per week, fixed *n*	4
Completed training sessions per week, median (IQR; range) ^a^	4 (3–4; 0–4)
Adherence rate, % ^a^	88%

^a^ Data were missing for 15 participants; ^b^ Data were missing for 12 participants; ^c^ Data were missing for 16 participants.

**Table 3:** Adherence and completion of the nutritional component.

	**Participants (*n* = 101)**
**Protein supplementation**	
Possible servings, fixed *n*	7
Completed servings, median (IQR; range)	7 (7–7; 0–7)
Adherence rate, %	93%
**Multivitamin supplementation**	
Possible doses, fixed *n*	7
Completed doses, median (IQR; range)	7 (7–7; 0–7)
Adherence rate, %	98%

## Data Availability

Data can be made available upon reasonable written request. The data are not publicly available due to the use of personal data.

## References

[1] Smits A., Smits E., Lopes A., Das N., Hughes G., Talaat A., Pollard A., Bouwman F., Massuger L., Bekkers R. (2015). Body mass index, physical activity and quality of life of ovarian cancer survivors: Time to get moving?. Gynecol. Oncol..

[2] Kathiresan A.S., Brookfield K.F., Schuman S.I., Lucci J.A. (2011). Malnutrition as a predictor of poor postoperative outcomes in gynecologic cancer patients. Arch. Gynecol. Obstet..

[3] Orekoya O., Samson M.E., Trivedi T., Vyas S., Steck S.E. (2016). The Impact of Obesity on Surgical Outcome in Endometrial Cancer Patients: A Systematic Review. J. Gynecol. Surg..

[4] Calle E.E., Rodriguez C., Walker-Thurmond K., Thun M.J. (2003). Overweight, obesity, and mortality from cancer in a prospectively studied cohort of U.S. adults. N. Engl. J. Med..

[5] Hami L.T., Lampe B., Mallmann P., Forner D.M. (2018). The Impact of Age on the Prognosis of Vulvar Cancer. Oncol. Res. Treat..

[6] Renehan A.G., Tyson M., Egger M., Heller R.F., Zwahlen M. (2008). Body-mass index and incidence of cancer: A systematic review and meta-analysis of prospective observational studies. Lancet.

[7] Schouten L.J., Goldbohm R.A., van den Brandt P.A. (2006). Anthropometry, physical activity, and endometrial cancer risk: Results from The Netherlands cohort study. Int. J. Gynecol. Cancer.

[8] Zhou W.L., Yue Y.Y. (2022). Trends in the Incidence of Vulvar and Vaginal Cancers with Different Histology by Race, Age, and Region in the United States (2001–2018). Int. J. Public Health.

[9] Koutoukidis D.A., Knobf M.T., Lanceley A. (2015). Obesity, diet, physical activity, and health-related quality of life in endometrial cancer survivors. Nutr. Rev..

[10] Bouwman F., Smits A., Lopes A., Das N., Pollard A., Massuger L., Bekkers R., Galaal K. (2015). The impact of BMI on surgical complications and outcomes in endometrial cancer surgery–An institutional study and systematic review of the literature. Gynecol. Oncol..

[11] Lin L.L., Brown J.C., Segal S., Schmitz K.H. (2014). Quality of life, body mass index, and physical activity among uterine cancer patients. Int. J. Gynecol. Cancer.

[12] Moore S.C., Gierach G.L., Schatzkin A., Matthews C.E. (2010). Physical activity, sedentary behaviours, and the prevention of endometrial cancer. Br. J. Cancer.

[13] Laky B., Janda M., Cleghorn G., Obermair A. (2008). Comparison of different nutritional assessments and body-composition measurements in detecting malnutrition among gynecologic cancer patients. Am. J. Clin. Nutr..

[14] Biller V.S., Leitzmann M.F., Sedlmeier A.M., Berger F.F., Ortmann O., Jochem C. (2021). Sedentary behaviour in relation to ovarian cancer risk: A systematic review and meta-analysis. Eur. J. Epidemiol..

[15] Iyer R., Gentry-Maharaj A., Nordin A., Burnell M., Liston R., Manchanda R., Das N., Desai R., Gornall R., Beardmore-Gray A. (2015). Predictors of complications in gynaecological oncological surgery: A prospective multicentre study (UKGOSOC-UK gynaecological oncology surgical outcomes and complications). Br. J. Cancer.

[16] Baldewpersad Tewarie N.M.S., van Driel W.J., van Ham M., Wouters M.W., Kruitwagen R., Participants of the Dutch Gynecological Oncology Collaborator Group (2021). Postoperative outcomes of primary and interval cytoreductive surgery for advanced ovarian cancer registered in the Dutch Gynecological Oncology Audit (DGOA). Gynecol. Oncol..

[17] de Groot J.J.A., Maessen J.M.C., Dejong C.H.C., Winkens B., Kruitwagen R., Slangen B.F.M., van der Weijden T., all the Members of the Study Group (2018). Interdepartmental Spread of Innovations: A Multicentre Study of the Enhanced Recovery After Surgery Programme. World J. Surg..

[18] Lucas A.R., Focht B.C., Cohn D.E., Klatt M.D., Buckworth J. (2018). Recruiting Endometrial Cancer Survivors to Studies Examining Lifestyle Behaviors and Quality of Life: Challenges Faced and Lessons Learned. J. Cancer Educ..

[19] Kokts-Porietis R.L., Elmrayed S., Brenner D.R., Friedenreich C.M. (2021). Obesity and mortality among endometrial cancer survivors: A systematic review and meta-analysis. Obes. Rev..

[20] El-Sherif A., El-Sherif S., Taylor A.H., Ayakannu T. (2021). Ovarian Cancer: Lifestyle, Diet and Nutrition. Nutr. Cancer.

[21] Johnston E.A., Ibiebele T.I., Friedlander M.L., Grant P.T., van der Pols J.C., Webb P.M., Ovarian cancer Prognosis and Lifestyle (OPAL) Study Group (2023). Association of Protein Intake with Recurrence and Survival Following Primary Treatment of Ovarian Cancer. Am. J. Clin. Nutr..

[22] van de Berg N.J., van Beurden F.P., Wendel-Vos G.C.W., Duijvestijn M., van Beekhuizen H.J., Maliepaard M., van Doorn H.C. (2023). Patient-Reported Mobility, Physical Activity, and Bicycle Use after Vulvar Carcinoma Surgery. Cancers.

[23] Banugo P., Amoako D. (2017). Prehabilitation. BJA Educ..

[24] Berkel A.E.M., Bongers B.C., Kotte H., Weltevreden P., de Jongh F.H.C., Eijsvogel M.M.M., Wymenga M., Bigirwamungu-Bargeman M., van der Palen J., van Det M.J. (2022). Effects of Community-based Exercise Prehabilitation for Patients Scheduled for Colorectal Surgery with High Risk for Postoperative Complications: Results of a Randomized Clinical Trial. Ann. Surg..

[25] Bruns E.R.J., van Rooijen S.J., Argillander T.E., van der Zaag E.S., van Grevenstein W.M.U., van Duijvendijk P., Buskens C.J., Bemelman W.A., van Munster B.C., Slooter G.D. (2019). Improving Outcomes in Oncological Colorectal Surgery by Prehabilitation. Am. J. Phys. Med. Rehabil..

[26] de Klerk M., van Dalen D.H., Nahar-van Venrooij L.M.W., Meijerink W., Verdaasdonk E.G.G. (2021). A multimodal prehabilitation program in high-risk patients undergoing elective resection for colorectal cancer: A retrospective cohort study. Eur. J. Surg. Oncol..

[27] Lambert J.E., Hayes L.D., Keegan T.J., Subar D.A., Gaffney C.J. (2021). The Impact of Prehabilitation on Patient Outcomes in Hepatobiliary, Colorectal, and Upper Gastrointestinal Cancer Surgery: A PRISMA-Accordant Meta-analysis. Ann. Surg..

[28] Waterland J.L., McCourt O., Edbrooke L., Granger C.L., Ismail H., Riedel B., Denehy L. (2021). Efficacy of Prehabilitation Including Exercise on Postoperative Outcomes Following Abdominal Cancer Surgery: A Systematic Review and Meta-Analysis. Front. Surg..

[29] Molenaar C.J.L., Minnella E.M., Coca-Martinez M., Ten Cate D.W.G., Regis M., Awasthi R., Martinez-Palli G., Lopez-Baamonde M., Sebio-Garcia R., Feo C.V. (2023). Effect of Multimodal Prehabilitation on Reducing Postoperative Complications and Enhancing Functional Capacity Following Colorectal Cancer Surgery: The PREHAB Randomized Clinical Trial. JAMA Surg..

[30] Bruns E.R., van den Heuvel B., Buskens C.J., van Duijvendijk P., Festen S., Wassenaar E.B., van der Zaag E.S., Bemelman W.A., van Munster B.C. (2016). The effects of physical prehabilitation in elderly patients undergoing colorectal surgery: A systematic review. Colorectal Dis..

[31] West M.A., Jack S., Grocott M.P.W. (2021). Prehabilitation before surgery: Is it for all patients?. Best. Pract. Res. Clin. Anaesthesiol..

[32] Dhanis J., Keidan N., Blake D., Rundle S., Strijker D., van Ham M., Pijnenborg J.M.A., Smits A. (2022). Prehabilitation to Improve Outcomes of Patients with Gynaecological Cancer: A New Window of Opportunity?. Cancers.

[33] Weggemans R.M., Backx F.J.G., Borghouts L., Chinapaw M., Hopman M.T.E., Koster A., Kremers S., van Loon L.J.C., May A., Mosterd A. (2018). The 2017 Dutch Physical Activity Guidelines. Int. J. Behav. Nutr. Phys. Act..

[34] van Exter S.H., Drager L.D., van Asseldonk M., Strijker D., van der Schoot N.D., van den Heuvel B., Verlaan S., van den Berg M.G.A. (2023). Adherence to and Efficacy of the Nutritional Intervention in Multimodal Prehabilitation in Colorectal and Esophageal Cancer Patients. Nutrients.

[35] Strijker D. Multimodal intensive prehabilitation in high impact surgery to reduce postoperative complications. *Int. Clin. Trial Regist. Platf.*
**2020**. https://trialsearch.who.int/Trial2.aspx?TrialID=NL8699.

[36] Riebe D., Franklin B.A., Thompson P.D., Garber C.E., Whitfield G.P., Magal M., Pescatello L.S. (2015). Updating ACSM’s Recommendations for Exercise Preparticipation Health Screening. Med. Sci. Sports Exerc..

[37] Bauer J., Capra S., Ferguson M. (2002). Use of the scored Patient-Generated Subjective Global Assessment (PG-SGA) as a nutrition assessment tool in patients with cancer. Eur. J. Clin. Nutr..

[38] Weijs P.J., Sauerwein H.P., Kondrup J. (2012). Protein recommendations in the ICU: G protein/kg body weight—Which body weight for underweight and obese patients?. Clin. Nutr..

[39] Spinhoven P., Ormel J., Sloekers P.P., Kempen G.I., Speckens A.E., Van Hemert A.M. (1997). A validation study of the Hospital Anxiety and Depression Scale (HADS) in different groups of Dutch subjects. Psychol. Med..

[40] Charlson M.E., Pompei P., Ales K.L., MacKenzie C.R. (1987). A new method of classifying prognostic comorbidity in longitudinal studies: Development and validation. J. Chronic Dis..

[41] IBM (2018). SPSS: Version 25 Pen Drive IBM SPSS Statistics 25.

[42] Diaz-Feijoo B., Agusti-Garcia N., Sebio R., Lopez-Hernandez A., Siso M., Glickman A., Carreras-Dieguez N., Fuste P., Marina T., Martinez-Egea J. (2022). Feasibility of a Multimodal Prehabilitation Programme in Patients Undergoing Cytoreductive Surgery for Advanced Ovarian Cancer: A Pilot Study. Cancers.

[43] Hertlein L., Zeder-Goss C., Furst S., Bayer D., Trillsch F., Czogalla B., Mahner S., Burges A., Rittler P. (2018). Peri-operative oral immunonutrition in malnourished ovarian cancer patients assessed by the nutritional risk screening. Arch. Gynecol. Obstet..

[44] Vidrine J.I., Sutton S.K., Wetter D.W., Shih Y.T., Ramondetta L.M., Elting L.S., Walker J.L., Smith K.M., Frank-Pearce S.G., Li Y. (2023). Efficacy of a Smoking Cessation Intervention for Survivors of Cervical Intraepithelial Neoplasia or Cervical Cancer: A Randomized Controlled Trial. J. Clin. Oncol..

[45] Bohlin K.S., Lofgren M., Lindkvist H., Milsom I. (2020). Smoking cessation prior to gynecological surgery-A registry-based randomized trial. Acta Obstet. Gynecol. Scand..

[46] Tonnesen H., Rosenberg J., Nielsen H.J., Rasmussen V., Hauge C., Pedersen I.K., Kehlet H. (1999). Effect of preoperative abstinence on poor postoperative outcome in alcohol misusers: Randomised controlled trial. BMJ.

[47] Chang M.C., Choo Y.J., Kim S. (2023). Effect of prehabilitation on patients with frailty undergoing colorectal cancer surgery: A systematic review and meta-analysis. Ann. Surg. Treat. Res..

[48] Daniels S.L., Lee M.J., George J., Kerr K., Moug S., Wilson T.R., Brown S.R., Wyld L. (2020). Prehabilitation in elective abdominal cancer surgery in older patients: Systematic review and meta-analysis. BJS Open.

[49] Guo Y., Ding L., Miao X., Jiang X., Xu T., Xu X., Zhu S., Xu Q., Hu J. (2022). Effects of prehabilitation on postoperative outcomes in frail cancer patients undergoing elective surgery: A systematic review and meta-analysis. Support. Care Cancer.

[50] Miralpeix E., Sole-Sedeno J.M., Rodriguez-Cosmen C., Taus A., Muns M.D., Fabrego B., Mancebo G. (2022). Impact of prehabilitation during neoadjuvant chemotherapy and interval cytoreductive surgery on ovarian cancer patients: A pilot study. World J. Surg. Oncol..

[51] Miralpeix E., Fabrego B., Rodriguez-Cosmen C., Sole-Sedeno J.M., Gayete S., Jara-Bogunya D., Corcoy M., Mancebo G. (2023). Prehabilitation in an ERAS program for endometrial cancer patients: Impact on post-operative recovery. Int. J. Gynecol. Cancer.

[52] Bland K.A., Krishnasamy M., Parr E.B., Mulder S., Martin P., van Loon L.J.C., Cormie P., Michael N., Zopf E.M. (2022). “I want to get myself as fit as I can and not die just yet”—Perceptions of exercise in people with advanced cancer and cachexia: A qualitative study. BMC Palliat. Care.

[53] Farrokhzadi L., Dhillon H.M., Goumas C., Young J.M., Cust A.E. (2016). Physical Activity Correlates, Barriers, and Preferences for Women with Gynecological Cancer. Int. J. Gynecol. Cancer.

